# DEVELOPMENT OF A TAILORED EDUCATIONAL PROGRAM TO ASSESS FALL RISK AND PREVENT FALLS FOR DIVERSE OLDER ADULTS.

**DOI:** 10.1093/geroni/igz038.3143

**Published:** 2019-11-08

**Authors:** Ladda Thiamwong, Norma E Conner

**Affiliations:** 1 College of Nursing, University of Central Florida, Orlando, Florida, United States; 2 University of Central Florida, Orlando, Florida, United States

## Abstract

Background: There is limited data on personal use fall prevention programs, and the relationship of race and ethnicity on fall risk awareness, personal beliefs, behavior change, and response to intervention. Objective: The aim of this study was to develop an educational program to prevent falls for ethnically diverse older adults. This program will be a culturally attuned program that values diversity and seeks to eliminate words and behaviors that might be discriminatory based on racial/ethnic or cultural identity. Methods: Three steps were used to develop the program: 1) constructing content domains; 2) generating the program draft; and 3) judging the program domain and content. The content domains were constructed based on data from a conventional content analysis of four focus groups from older participants (n=28) and their family caregivers (n=4), and individual in-depth interviews from health care providers (n=8). We generated the program outline with three response choices. Eight older participants and two health care providers rated it. Results: The program consisted of risk assessment, outreaching and raising awareness and knowledge. Risk assessment: all participants suggested that risk assessment should consists of objective and subjective measures. Outreaching: participants agreed that group-teaching and individual learning by peer coaching based on their culture, new blasts, brochures, and family-based approaches were the best outreaching methods that they preferred. They identified that raising awareness and knowledge should include the following topics: performing physical activity with fall risk awareness, medication management, visual care, behavioral adaptation with appropriate accessories /equipment, and environmental safety.

